# Artificial Vision: The High-Frequency Electrical Stimulation of the Blind Mouse Retina Decay Spike Generation and Electrogenically Clamped Intracellular Ca^2+^ at Elevated Levels

**DOI:** 10.3390/bioengineering10101208

**Published:** 2023-10-16

**Authors:** Lucia Peiroten, Eberhart Zrenner, Wadood Haq

**Affiliations:** Neuroretinal Electrophysiology and Imaging, Institute for Ophthalmic Research, Eberhard-Karls-Universität Tübingen, 72076 Tübingen, Germany; lucia.peiroten@uni-tuebingen.de (L.P.);

**Keywords:** retinal degeneration, rd1 mouse retina, vision rescue, artificial vision, electrical stimulation, calcium imaging, MEA recordings, neuronal desensitization

## Abstract

Background: The electrical stimulation (stim) of retinal neurons enables blind patients to experience limited artificial vision. A rapid response outage of the stimulated ganglion cells (GCs) allows for a low visual sensation rate. Hence, to elucidate the underlying mechanism, we investigated different stim parameters and the role of the neuromodulator calcium (Ca^2+^). Methods: Subretinal stim was applied on retinal explants (blind *rd1* mouse) using multielectrode arrays (MEAs) or single metal electrodes, and the GC activity was recorded using Ca^2+^ imaging or MEA, respectively. Stim parameters, including voltage, phase polarity, and frequency, were investigated using specific blockers. Results: At lower stim frequencies (<5 Hz), GCs responded synaptically according to the stim pulses (stim: biphasic, cathodic-first, −1.6/+1.5 V). In contrast, higher stim frequencies (≥5 Hz) also activated GCs directly and induced a rapid GC spike response outage (<500 ms, MEA recordings), while in Ca^2+^ imaging at the same frequencies, increased intracellular Ca^2+^ levels were observed. Conclusions: Our study elucidated the mechanisms involved in stim-dependent GC spike response outage: sustained high-frequency stim-induced spike outage, accompanied by electrogenically clamped intracellular Ca^2+^ levels at elevated levels. These findings will guide future studies optimizing stim paradigms for electrical implant applications for interfacing neurons.

## 1. Introduction

Blinding diseases, such as hereditary retinitis pigmentosa, affect millions of people worldwide [1,2,3]. The concept of microchip implants, which excite neurons through stim [4,5], has been successfully evaluated in clinical trials that have partially restored the vision of blind patients (subretinal implants [6,7,8,9], epiretinal implants [10,11], and cortical implants [12,13,14]). In clinical trials, blind patients implanted with these microelectronics accomplished daily life tasks, such as recognizing simple shapes, reading large letters, and combining them into words [9,15,16]. In advanced mobility tests, some patients could track a line on the floor, follow a walking person, identify obstacles, and even evaluate distances in both daylight and nighttime conditions [17,18,19].

Regarding performance, artificial vision technology still faces limitations in terms of spatial and, in particular, temporal resolution. For most patients, the visual sensation rate was limited to 0.3 s at a 3 Hz stim frequency; higher stim frequencies led to vision fading [15,20,21]. This phenomenon was investigated in animal experiments, which showed that the fading of vision experienced by patients originates in the stimulated retina at the cellular level. Thereby, the high-frequency stim of retinal explants leads to an outage of ganglion cell (GC) responses [7,22,23,24,25]. It has been proposed that the desensitization of membrane channels facilitates the response outage in neurons [26,27,28]. However, no conclusive evidence has yet been presented describing the underlying mechanism for retinal GC response outage.

In our previous studies investigating the outer retina of a *rd1* mouse model for retinal degeneration [29], we observed that cellular calcium (Ca^2+^) responses are modulated by the strength of the applied stim. Hence, in the present study, we thoroughly investigated the role of Ca^2+^ in shaping GC responses. Different stim parameters were investigated, including voltage (0.5–2.5 V), polarity (anodic- or cathodic-first), the distance of the stim electrode to the reference electrode, and frequencies (various frequencies up to 50 Hz for 90 s). We utilized multimodal methods to record GC responses (Ca^2+^ imaging and multielectrode array (MEA)) and applied specific synaptic and channel blockers to determine the role of Ca^2+^ in the modulation of GC responses.

The temporal correlation of GC Ca^2+^ responses and spike trains revealed interesting yet undescribed findings. At high stim frequencies (sustained application), while the GC spike responses were absent, the intracellular Ca^2+^ levels were remarkably elevated. We concluded that high-frequency stim prevented electrogenically cellular Ca^2+^ extrusion and thereby clamped intracellular Ca^2+^ at high levels with probable electrophysiological implications for the further generation of GC spikes during ongoing sustained stim.

These findings are interesting for studies optimizing stim paradigms for artificial vision to counter vision fading and, overall, for electrical implants’ neural interfaces.

## 2. Materials and Methods

### 2.1. Animals

This study used a blind *rd1* (C3H Pde6b^rd1/rd1^) mouse model (age: 19–39 days; n = 11) for retinal degeneration with primary rod degeneration and secondary cone dystrophy [30]. The animals were housed under standard light conditions, had free access to food and water, and were used irrespective of gender. The animals were anesthetized in a carbon dioxide atmosphere and were immediately sacrificed by cervical dislocation.

### 2.2. Tissue Preparation and Loading

After enucleating both eyes, the retinas were isolated in artificial cerebrospinal fluid (ACSF) containing (in mM) 125 NaCl, 26 NaHCO_3_, 2.5 KCl, 2 CaCl_2_, 1 MgCl_2_, 1.25 NaH2PO_4_, and 20 glucose (obtained from Sigma-Aldrich, Darmstadt, Germany). A pH of 7.4 was maintained by perfusing it with carbogen (95% CO_2_/5% O_2_). To minimize the number of animals used, the explanted retina was cut in half, and each half was used for recording. For Ca^2+^ imaging, the GCs were loaded by applying electroporation with fluorescent Ca^2+^ dye Oregon Green 488 BAPTA-1 (OGB-1, Sigma-Aldrich, Darmstadt, Germany). The electroporation method for bulk loading described by Brigman and Euler [31] was adapted for the stim of *rd1* retina. For loading (5 µM, diluted in ACSF), 20 monophasic pulses of 7 V with a pulse duration of 100 ms were applied at 1 Hz (platinum disk electrode on Petri dish, 5 mm Ø, and 1 mm height frame; 4 mm Ø platinum disk electrode on the tip of the stick, Xceltis GmbH, Heidelberg, Germany). After the electroporation process, the retina was incubated in ACSF for 1 h to recover before being transferred to the MEA recording chamber. Prior to recording, the retina rested for an additional 30 min on the MEA electrode field with ACSF perfusion (1 mL/min).

### 2.3. Electrical Stimulation and Recording

**Figure 1:**
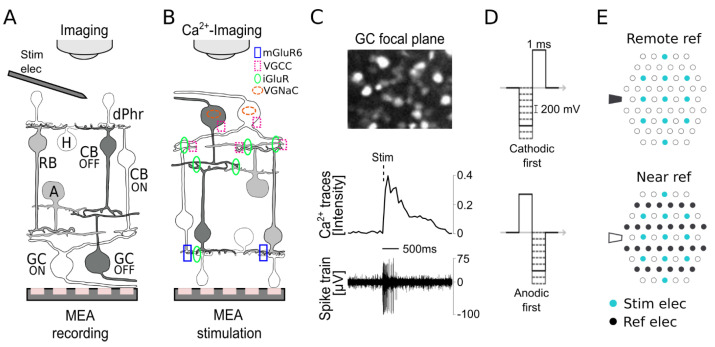
**Subretinal electrical stimulation and ganglion cell recordings.** Electrical stimulation (stim) of the blind mouse (rd1) retina in two different subretinal configurations: (**A**) Stim via a single microelectrode and recordings of retinal ganglion cell (GC) spikes using a multielectrode array (MEA). The stim electrode (elec) was brought into contact with the outer retina under microscopic control. (**B**) MEA stim of the outer retina and recording of GC activity via calcium imaging (Ca^2+^ imaging). The symbols indicate the glutamate receptors (metabotropic glutamate receptor 6 (mGluR6) and ionic glutamate receptor (iGluR)) and the voltage-gated channels (voltage-gated Ca^2+^ channels (VGCCs) and voltage-gated sodium channels (VGNaCs)). (**C**) Ca^2+^ imaging of GCs loaded with the fluorescent Ca^2+^ indicator OGB-1 (upper panel). Temporal correlation of a representative GC Ca^2+^ trace (Ca^2+^ imaging) and spike train (MEA recording) to the subretinal biphasic cathodic-first stim pulse (−1.6/+1.5 V). (**D**) Pulse profile diagram of the applied biphasic stim in cathodic-first (upper panel) or anodic-first (lower panel) modes. The positive phase was kept static at a certain voltage (solid line), while the negative phase was modulated (dashed line) in 200 mV subvoltage steps. (**E**) MEA layout of the spatial configuration of the stim and the reference (ref) elec (MEA dimensions: diameter 10 µm and spacing 40 µm; remote ref (upper panel) and near ref (lower panel). Abbreviations of the retinal cells: dPhr: degenerated light-insensitive photoreceptors, H: horizontal cell, RB: rod bipolar cell, CB OFF: OFF-type cone bipolar cell, CB ON: ON-type bipolar cell, GC ON: ON-type GC, and GC OFF: OFF-type GC.

The retina was placed in an MEA system (USB-MEA60-Up-BC-System-E, Multi-Channel Systems (MCS), Reutlingen, Germany) equipped with a 60HexaMEA40/10iR-ITO-pr (MCS, Reutlingen, Germany) consisting of 59 individual electrodes with a diameter of 10 µm in a 40 µm distance and an internal reference electrode. The built-in temperature system of the MEA was set to 32 °C. The mean impedance of the MEA electrodes was 1.0 MΩ (NanoZ v 1.2, MCS, Reutlingen, Germany). Either the subretinal stim was applied with an MEA and the GC activity was recorded via Ca^2+^ imaging, or the stim was applied using a single metal electrode (platinum/iridium microelectrodes, tip ~3 µm, ~7 kOhms, MicroProbes, Gaithersburg, MD, USA), and the GC activity was recorded with an MEA (cf. Figure 1A,B). The external stim microelectrode was positioned under the microscope (20 X) using a micromanipulator (5171, Eppendorf, Hamburg, Germany). Ca^2+^ imaging recordings were carried out using an upright fluorescence microscope (BX50WI, Olympus, Germany) equipped with a 20 X water immersion objective (LUMPlan FL, 40X/0.80W, ∞/0, Olympus, Hamburg, Germany), a polychromator (VisiChrome, Visitron Systems, Puchheim, Germany), and a CCD camera (RETIGA-R1, 1360 × 1024 pixels, 16 bit). Image stacks of the OGB-1 fluorescence were acquired at 50 Hz (470 nm excitation; Olympus U-MNU filter set, 20 ms exposure time, 8-pixel binning) using VisiView software (V 3.1, Visitron Systems, Puchheim, Germany). For each experiment, an image of the electrodes (MEA and single microelectrode) and the retina was taken to correlate with the spatial distance of the stimulating electrode and responsive GCs. GC spike activity was recorded via the MEA system at 20 kHz raw data sampling. Ca^2+^ imaging and MEA recordings were both synchronized with the stim using a trigger, which was controlled by the recording protocol set within the MC_Rack software (v 4.6.2, MCS, Reutlingen, Germany) and the digital input–output box (MCS, Reutlingen, Germany). For each experiment (Ca^2+^ imaging or MEA), 20 s of control was recorded before stim; then, the application of the stim (+trigger detection) and 20 s of the post-stim period were recorded. When applying stim with an MEA, MEA_select (MCS, Reutlingen, Germany) was used to define the stim and reference electrodes (Figure 1C). The stim protocols were generated with an STG2008 device (MCS, Reutlingen, Germany) using MC Stimulus II software (v 3.4.4, MCS, Reutlingen, Germany). Stim protocols were defined as follows: (a) To find the best-performing stim paradigm, four stim paradigms were designed (cathodic^remote^, cathodic^near^, anodic^remote^, and anodic^near^; cf. Figure 1D,E) by modulating three parameters: (i) the polarity of the biphasic stim pulses (cathodic-first or anodic-first); (ii) the reference electrode configuration, either a single remote reference electrode (using an internal reference electrode of the MEA) or multiple reference electrodes close to the stim electrodes (MEA electrodes used as references) (cf. Figure 1E); and (iii) a voltage ramp grouped in voltage blocks of 0.5, 1.0, 1.5, 2.0, and 2.5 V of biphasic pulses (1 ms/phase). Within each voltage block, only the cathodic phase varied in −0.2 V subvoltage steps, while the anodic phase remained constant. In total, 54 subvoltages (cf. Figure 1D) were applied at 0.05 Hz (20 s interval). Only Ca^2+^ imaging was applied to record the GC activity for these experiments. (b) To investigate the role of Ca^2+^ in GC response outage, we applied a sustained 90 s stim at various frequencies (0.5, 1.5, 3, 5, 10, 20, and 50 Hz), using the stim parameters (cathodic^near^ and −1.6/+1.5 V) established in (a). Here, both Ca^2+^ imaging and MEA were used to record GC activity.

### 2.4. Data Analysis

#### 2.4.1. Ca^2+^ Imaging

Regions of interest were set manually on GCs in the proximity of the stim electrodes, and the corresponding Ca^2+^ traces were extracted using ImageJ software (version 1.53n, https://imagej.nih.gov/ij/ (accessed on 7 September 2021). The Ca^2+^ δ-amplitudes were obtained by calculating the difference between the Ca^2+^ signal baseline (averaged from the pre-stim phase) and the stim-evoked Ca^2+^ amplitude (identified by the stim trigger mark). The signal decay (τ) was calculated to estimate the refractory time of the GC’s Ca^2+^ response (66% signal drop in seconds). The Ca^2+^ data were normalized against the highest occurring Ca^2+^ value in the respective dataset. Further data analysis and the production of plots were carried out using custom-developed scripts (MATLAB, 2020b, The MathWorks, Natick, MA, USA). For statistical analysis of the drug data, one-way ANOVA followed by Dunnett’s multiple-comparison test was applied, and for the comparison of the frequencies (ctr), the Wilcoxon–Mann–Whitney test was utilized to compare them pairwise with the next higher frequency (MATLAB, The MathWorks, Natick, MA, USA).

#### 2.4.2. MEA Recordings

For spike extraction, a high-pass filter was applied to the raw data (200 Hz, Butterworth 2nd order; MC Rack MCS, Reutlingen, Germany). The filtered files were exported to HDF5 file format with the MC DataManager (v1.14.4.22018, MCS, Reutlingen, Germany) and were read in MATLAB using the McsMatlabDataTools (MCS, Reutlingen, Germany) software bundle. Custom-developed MATLAB scripts were used for further data processing [25]. A static threshold of ±17 µV was set for spike detection, and only bimodal-shaped spikes were selected for the analysis. The stim-evoked spike responses were detected according to the trigger time stamp, and the responses of single cells recorded on each electrode were isolated. The GC spike activity was presented in a histogram (rate: spike count per 50 ms bin). Our study focused on cell-type independent response outage; hence, cell sorting was not used to discriminate between major GC types (ON, OFF, and ON–OFF). The image editing software Inkscape (v1.2.2, www.inkscape.org (accessed on 27 December 2022) was used to compile the presented figures.

### 2.5. Pharmacology

Pharmaceutical agents were directly injected into the MEA chamber, and the retina was incubated for 30 min before recording. We used (in µM) 100 L-AP4 (mGluR6 agonist; L-2-amino-4-phosphonobutyric acid), 20 CNQX (AMPA/kainite-type GluR antagonist; 6-Cyano-7-nitroquinoxaline-2,3-dione) obtained from TocrisBioScience (Bristol, UK); 100 verapamil (L-type voltage-gated Ca^2+^ channel (VGCC) blocker) obtained from Sigma-Aldrich (Darmstadt, Germany); and 30 TTX (sodium channel blocker; tetrodotoxin) obtained from Carl Roth (Karlsruhe, Germany).

## 3. Results

### 3.1. Subretinal Electrical Stimulation and Ca^2+^ Imaging of Ganglion Cell Responses

To investigate the role of Ca^2+^ in the response outage of retinal GCs, we established a novel experimental method combining subretinal stim using MEAs (Figure 1B) with the simultaneous recording of GC activity using Ca^2+^ imaging. Therefore, we adapted the retinal bulk electroporation of the OGB-1 Ca^2+^ indicator principally used for two-photon Ca^2+^ imaging [31,32]. However, in our approach, we electroporated the GCs of the photoreceptor-degenerated retina (*rd1* mouse) with OGB-1 and recorded (brightfield confocal imaging) the GC Ca^2+^ responses elicited by stim. On the one hand, this method allowed us to assess the stim-dependent GC activity with high spatial resolution at the cellular level (Figure 1C); on the other hand, the spatial projection of the stim electrodes (focus through the retina) on the GC focal-plane image enabled us to correlate the stim electrode and the responding cell.

Moreover, the stimulus-dependent correlation of the GC spike recording (elicited with a single metal electrode and MEA recordings, Figure 1A) with Ca^2+^ signals showed that the spikes occurred during the increase in the Ca^2+^ transient (Figure 1C) and were absent during the significantly longer signal recovery phase, which is crucial for the generation of subsequent responses. Furthermore, in conjunction with retina-specific pharmaceuticals, the methods complemented each other as a powerful tool for our retinal function investigation (Figure 1B).

### 3.2. Variation in the Electrical Stimulation Paradigm Modulates the Strength of the Ganglion Cell Ca^2+^ Responses

**Figure 2:**
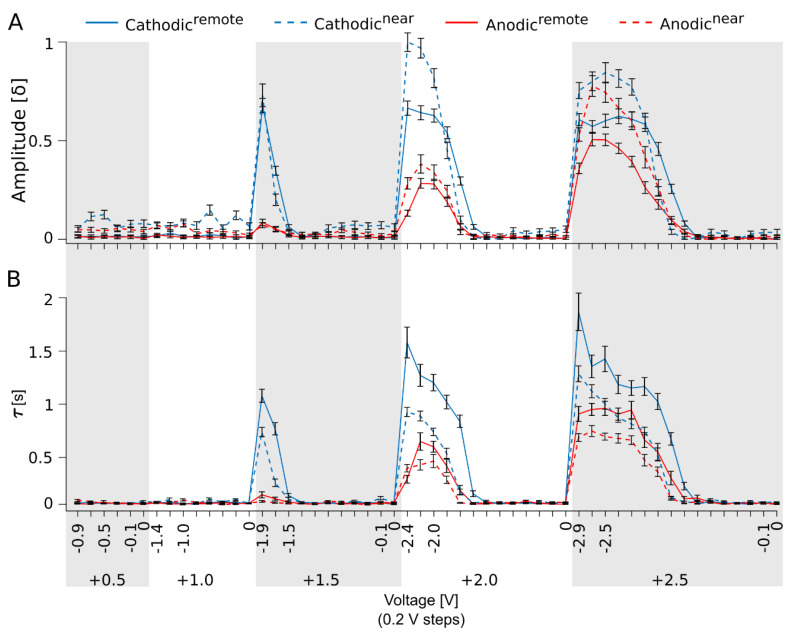
**Evaluation of ganglion cell Ca^2+^ responses elicited through the modulation of stimulation paradigms.** Presentation of ganglion cell (GC) Ca^2+^ responses: (**A**) Amplitude (δ; indicating Ca^2+^ intrusion) and (**B**) decay time (τ, estimating Ca^2+^ extrusion at 66% in seconds) evoked under four different stim paradigms by modulation of three parameters: (1) biphasic pulse: cathodic-first (blue line) or anodic-first (red line) (cf. Figure 1D, each phase of 1 ms duration); (2) modulation of the biphasic pulse (cf. Figure 1D), with the anodic phase kept static to define the five voltage blocks (+0.5, +1.0, +1.5, +2.0, and +2.5 V) and only the cathodic phase modulated in −0.2 V subvoltage steps (in case of odd voltage blocks additionally a stim at −0.1 V was carried out); and (3) reference electrode configuration: remote (solid line) or near (dashed line) (cf. Figure 1E). N = 5 retina, m = 6 cells, stim pulse interval 20 s, and 5 repetitions of each pulse. Error bars indicate ± SEM.

To achieve our primary goal of investigating the dependence of the GC response outage on high-frequency stim, we first systematically evaluated the best-performing stim paradigm for Ca^2+^ imaging. We designed four different stim paradigms (Figure 2: cathodic^remote^, cathodic^near^, anodic^remote^, and anodic^near^) by modulating three parameters: (i) the phase of the biphasic pulse: cathodic- or anodic-first (see also Figure 1D, 1 ms/phase); (ii) the voltage modulation of the biphasic pulse (see also Figure 1D for pulse profile), through which the anodic (positive) phase was kept static, defining the five voltage blocks (+0.5, +1.0, +1.5, +2.0, and +2.5 V), and only the cathodic (negative) phase was modulated in −0.2 V steps; and (iii) the reference electrode configuration: remote or near (Figure 1E). These experiments were carried out at a low stim frequency (0.05 Hz) to determine the stim-dependent Ca^2+^ dynamics in the GCs of the blind *rd1* mouse.

In total, 54 subvoltage steps were applied. Remarkably, the GC did not respond to each subvoltage step (Figure 2A). To stay in the context of the project’s scope, we evaluate and discuss only the most significant response events relevant to frequency modulation experiments.

The evaluation of GC Ca^2+^ responses’ δ-amplitude (Figure 2A) determined that, overall, the cathodic-first (voltage polarity: −/+) stim paradigm performed better in subretinal stim than the anodic-first (voltage polarity: +/−) stim category. The first visible responses at lower voltage blocks were induced by cathodic-first stim (δ-amplitude: at −0.5/+0.5 V = 0.13 ± 0.02 and at −0.6/+1.0 V = 0.15 ± 0.03). With an increase in stim strength, larger cathodic-first than anodic-first response amplitudes were observed (−1.9/+1.5 V = 0.73 ± 0.06, 10.43-fold larger than +1.5/−1.9 V, and −2.4/+2.0 V = 1.0 ± 0.05, 3.57-fold larger than +2.0/−2.4 V). At the highest applied voltage, response saturation, rather than an increase in amplitude, was observed. Moreover, a minor difference between anodic-first and cathodic-first stim was found (−2.5/+2.3 V = 0.81 ± 0.04; 1.22-fold larger than +2.3/−2.5 V). The gain in responses to anodic-first stim at these high voltages indicates a direct activation of GCs via voltage-gated channels (VGCs), rather than network-mediated activation (as seen in the previous lower stim). Additionally, the distance of the stim electrode to the reference was relevant; in the context of subclasses, cathodic^near^ performed overall up to 1.5-fold better than cathodic^remote^, and anodic^near^ performed better than anodic^remote^ (1.3-fold).

We extended our investigation to evaluate the Ca^2+^ signal’s decay time τ (66% signal drop in seconds), which indicates the cell’s recovery time. This parameter is crucial when investigating higher stim frequencies regarding the overlap of following stim responses. The τ value of anodic-first responses was naturally smaller than the cathodic-first because the generated Ca^2+^ amplitudes were also smaller. However, in the same category of cathodic-first stim, the cathodic^near^ constellation performed better than cathodic^remote^ (1.46- to 1.85-fold smaller). Overall, cathodic-first, particularly cathodic^near^, performed best in subretinal stim compared with others, generating the largest Ca^2+^ amplitudes with much faster τ.

Based on the given data, as an optimal stim pulse for further investigation of the frequency domain, a cathodic-first pulse (−1.6/+1.5 V, cathodic^near^) was selected: optimal amplitude with relatively low decay time (−1.7/+1.5 V: delta peak = 0.26 ± 0.03; τ = 0.26 ± 0.04 s).

### 3.3. The Characteristics of Ganglion Cell Ca^2+^ Responses Are Modulated by Electrical Stimulation Frequencies

Investigating the role of Ca^2+^ in the dependence of the GC response outage on stim frequency, we subretinally stimulated the retinal tissue for 90 s at various frequencies (0.5, 1.5, 3, 5, 10, 20, and 50 Hz; cathodic-first pulse, −1.6/+1.5 V, cathodic^near^).

**Figure 3:**
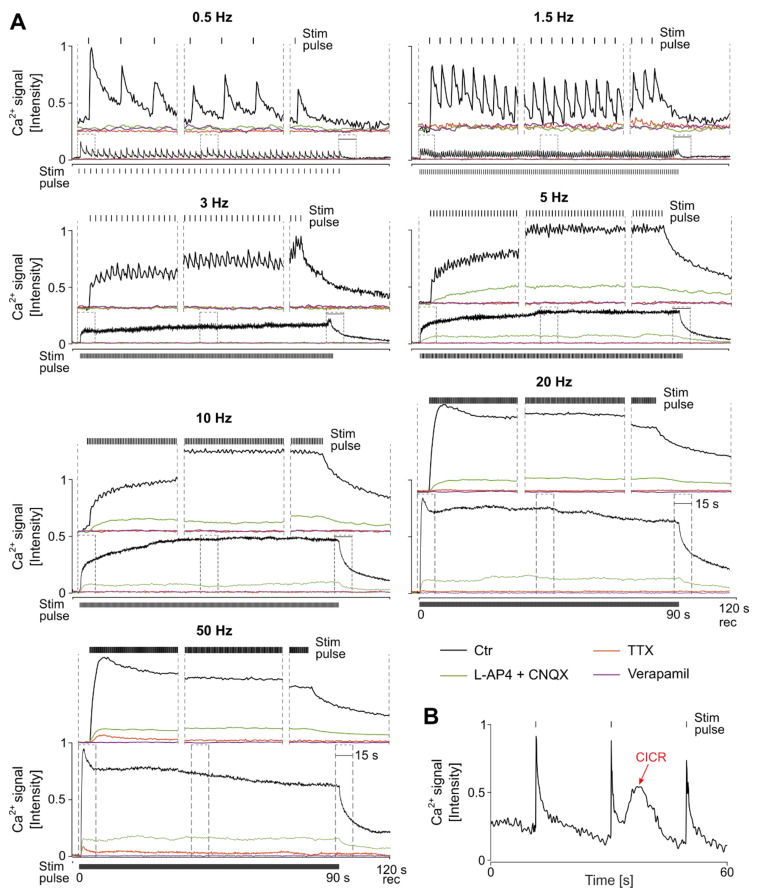
**Stimulation frequency-dependent modulation of retinal ganglion cell responses:** (**A**) Ca^2+^ imaging recordings of ganglion cells (GCs, mean of n = 6 retinas, m = 6 cells) elicited via sustained electrical stimulation (stim; 90 s) at various frequencies (0.5, 1.5, 3, 5, 10, 20, and 50 Hz; biphasic stimulus: −1.6/+1.5 V). Temporal magnification of the response’s initial, middle, and recovery phases is indicated in the inset boxes (15 s resolution). Experimental recording conditions: control (ctr) and in the presence of pharmaceuticals (cf. Figure 1 for site of action): mGluR6 agonist L-AP4, iGluR antagonist CNQX, VGNaC antagonist TTX, and VGCC blocker verapamil. (**B**) Representative Ca^2+^-induced Ca^2+^-release (CICR) events (black) were rarely recorded in a GC during subretinal stim.

Lower stim frequencies (0.5 and 1.5 Hz) generated typical Ca^2+^ signals (Figure 3, ctr recording), with a transient characteristic (Ca^2+^ influx) correlating temporally with the applied stim pulse and the following recovery (Ca^2+^ efflux). While at 0.5 Hz, the Ca^2+^ signals were clearly separated (2 s interval) at 1.5 Hz (0.7 s interval), the following Ca^2+^ signal continued to the end of the recovery phase of its previous signal.

Upon higher stim frequencies (>1.5 Hz), the Ca^2+^ response amplitudes became smaller and eventually started to diminish. Hence, for a better evaluation and understanding, we discriminated between the responses’ initial, middle, and recovery phases (Figure 3, inset of 15 s). Within the initial phase at higher frequencies (3, 5, 10, 20, and 50 Hz), the GCs responded to the very first pulses with a transient Ca^2+^ signal (pulse interval: 333, 200, 100, 50, and 20 ms), and Ca^2+^ responses increased with the increase in stim frequency (values for first peak to respective frequencies: 0.15 ± 0.03, 0.11 ± 0.02, 0.14 ± 0.01, 0.18 ± 0.01, 0.24 ± 0.02, 0.88 ± 0.13, and 1.0 ± 0.02). With progressing stim, in the middle phase at 3 and 5 Hz stim, the observed Ca^2+^ transients correlated with the stim pulses, but the amplitudes were significantly smaller and “resided” on elevated intracellular Ca^2+^ levels. These data indicate that Ca^2+^ responses did not have enough time to recover; hence, the subsequent responses overlapped with the previous ones. The resulting elevated Ca^2+^ level of the middle phase was approximately aligned with the response amplitude of the very first pulses within the initial phase (3 Hz = 0.02 units smaller and 5 Hz = 0.06 units smaller). At comparatively higher frequencies (10, 20, and 50 Hz), no Ca^2+^ peaks were observed to be temporally correlated with applied pulses, but again, the intracellular Ca^2+^ was elevated. The established Ca^2+^ plateau was level with the initial response (10 Hz = 0.19, 20 = 0.20, and 50 = 0.35 units smaller) and was maintained during the stim period. At all frequencies, the elevated intracellular Ca^2+^ level recovered immediately post stimulus.

Additionally, it is worth mentioning that stim-evoked Ca^2+^-induced Ca^2+^ release (CICR) events were rarely observed (Figure 3B). CICR events were relatively slow and therefore differed significantly in their characteristics from Ca^2+^ signals mediated through voltage-gated sodium channels (VGNaCs; spikes, rapid, and transient). Hence, Ca^2+^ traces exhibiting CICRs were identified easily and not considered for stim/amplitude evaluation.

Overall, the stim frequency shaped the cellular Ca^2+^ transient (intrusion) and its recovery (extrusion). With the increase in stim frequencies, larger Ca^2+^ response transients were observed, and the GC Ca^2+^ responses melted, forming a plateau. The intracellular Ca^2+^ remained clamped at elevated levels during the stim period and recovered immediately after stim.

### 3.4. Stimulation Frequency-Dependent Activation of Ganglion Cells: Network-Mediated or Direct Activation

To assess whether the subretinally applied high stim frequencies (Figure 3) activated the GCs through the retinal network (synaptic) or directly via the GCs’ VGCs, specific pharmaceuticals were utilized (cf. Figure 1B) during Ca^2+^ imaging recording.

**Figure 4:**
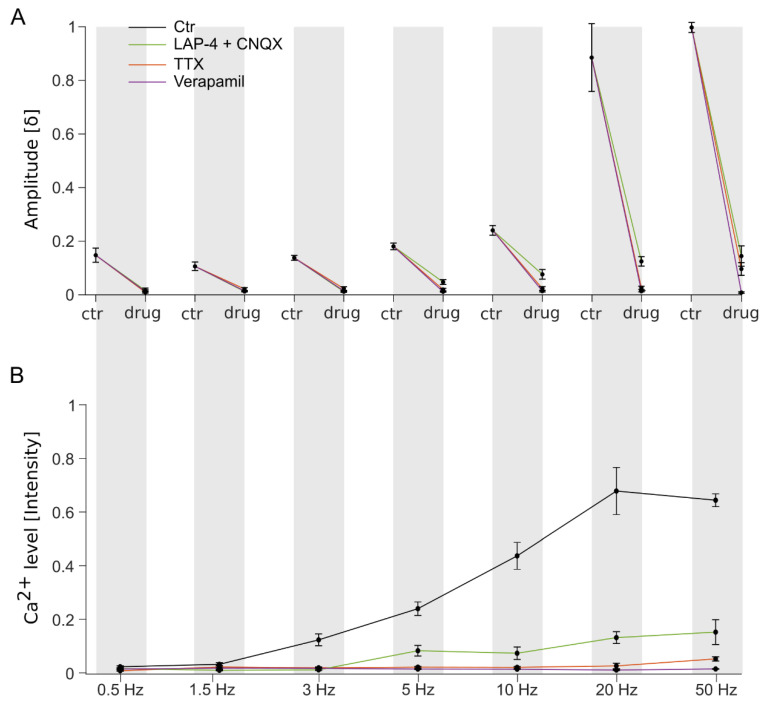
**Quantification of the ganglion cell Ca^2+^ responses modulated by various frequencies and pharmaceuticals.** Stimulation (stim) frequency (*x*-axis: 0.5, 1.5, 3, 5, 10, 20, and 50 Hz; biphasic stimulus: −1.6/+1.5 V)-dependent modulation of ganglion cell (GC responses under control (ctr, black) and in the presence of pharmaceuticals (colored) (cf. Figure 1 for site of action): mGluR6 agonist L-AP4, iGluR antagonist CNQX, VGNaC antagonist TTX, and VGCC blocker verapamil. Quantification of (**A**) **δ**-amplitude (*y*-axis normalized, first response peak within early response phase; see Figure 3), and (**B**) estimation of elevated Ca^2+^ levels (*y*-axis normalized, average Ca^2+^ level of middle recording phase; see Figure 3). For each condition, n = 6 retina, m = 6 cells. Error bars indicate ± SEM. Statistical analysis is provided in Table S1.

Lower stim frequencies (0.5, 1.5, and 3 Hz) promoted network-mediated responses (dPhr => BC => GC or BC => GC), which were diminished by ~95% in the presence of the glutamate receptor blockers (mGluR6 agonist LAP-4 and iGluR antagonist CNQX; traces shown in Figure 3, quantification shown in Figure 4, and statistics shown in Table S1). Additionally, our assessment was supported by the inhibition of the network’s synaptic transmission (VGCC antagonist verapamil) and the GC responses (VGNaC antagonist TTX), showing no GC activity at all after the respective inhibition (~95% response drop).

GC Ca^2+^ responses were recorded at higher stim frequencies (5, 10, 20, and 50 Hz) despite the blockage of the glutamatergic synaptic pathway (initial phase: ~ 88% decline; middle phase: ~ 77% decline). In contrast, the antagonization of VGCC and VGNaC silenced the GC responses entirely (average initial and middle phase: 97% decline), suggesting the direct electrical activation of the GCs via VGC.

Overall, higher stim frequencies penetrated the tissue deeper during subretinal stim. Mainly, the GCs were activated synaptically (~88%) and directly (~12%) over the GCs’ VGCCs and VGNaCs.

### 3.5. Continuous High-Frequency Electrical Stimulation Does Not Generate Pulse-Correlated Spikes in Ganglion Cells but Clamps Intracellular Ca^2+^ at Elevated Levels

**Figure 5:**
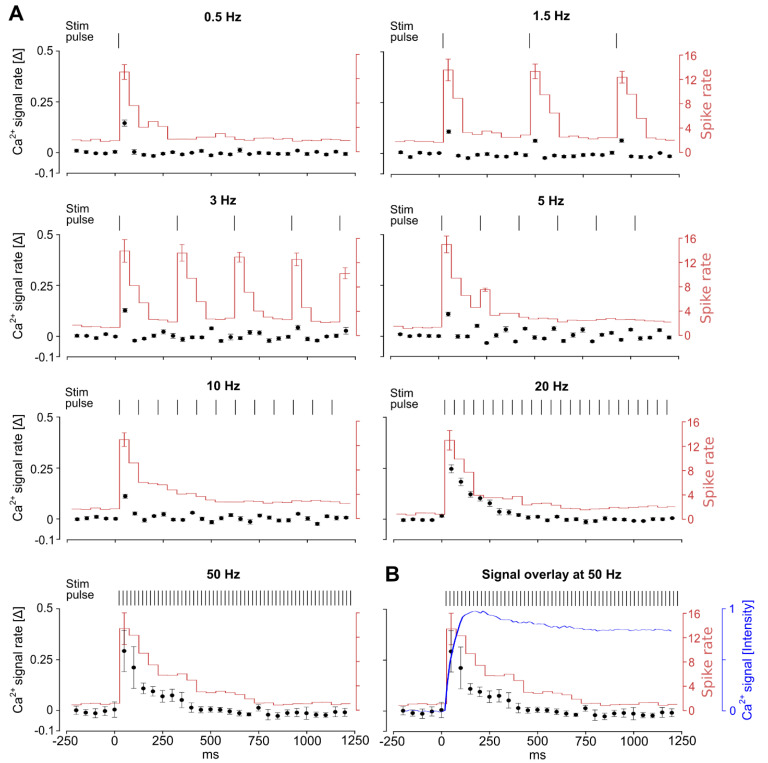
**Temporal course of ganglion cell responses in dependency of stimulation frequency.** (**A**) Correlation of ganglion cell (GC) responses to electrical stimulation (stim) pulses at various stim frequencies (0.5, 1.5, 3, 5, 10, 20, and 50 Hz; biphasic cathodic-first stimulus: −1.6/+1.5 V) within the early response phase (1250 ms, cf. Figure 3). Ca^2+^ imaging (left *y*-axis, black dots): rate (∆) Ca^2+^ signal (rate of signal change/50 ms bin); MEA recording (right *y*-axis, red solid line): spike rate (spike count/50 ms bin). (**B**) Representative overlay of data at 50 Hz stim: Ca^2+^ trace (blue, intensity, cf. Figure 3), spike rate, and Ca^2+^ signal rate. Ca^2+^ data: n = 6 retina, m = 6 cells, and MEA data: n = 5 retina. Error bars indicate ± SEM (only for the largest most significant data points in MEA recordings).

To elucidate the full extent of the observed elevated intracellular Ca^2+^ levels, we correlated GC spike recordings (MEA recordings, Figure 1A) with Ca^2+^ responses (Ca^2+^ imaging, Figure 1B) at each respective frequency (Figure 5A). We analyzed the runoff of the GC responses within the initial stim phase (1250 ms), considering the spike rate, which indicates response outage (spike count/50 ms, red line, right axis), and the Ca^2+^ rate, which indicates stim-induced Ca^2+^ intrusion (∆-Ca^2+^/50 ms, with ∆ = [Ca^2+^]_t_ − [Ca^2+^]_t-1_, black dots, left axis). Lower stim frequencies (0.5, 1.5, and 3 Hz) elicited GC spike responses and Ca^2+^ signals in accordance with the respective stimulus pulse (Ca^2+^ traces, Figure 3; temporal analysis, Figure 5A). For higher stim frequencies (5, 10, 20, and 50 Hz), it was determined that only stim pulses within the first 50 ms yielded the largest increase in cellular Ca^2+^ (Ca^2+^ traces, Figure 3; temporal analysis, Figure 5A: 0.10 ± 0.01, 0.11 ± 0.01, 0.25 ± 0.02, and 0.29 ± 0.10 units), which correlated temporally with the highest observed spike rates (15.14 ± 1.4, 7.71 ± 0.27, 12.78 ± 1.12, 13.34 ± 1.61, and 13.49 ± 2.57). Over the time course, GC spike responses appeared only within the first 500 ms and occasionally (noise level) for the remaining stim period (Figure 5A, on average ~ 3.5 spikes/50 ms), indicating a GC response outage. During the same stim timeline, in Ca^2+^ recordings, either further cellular Ca^2+^ influx was detected (Figure 5A; Ca^2+^ rate lies by ctr values before stim) or extrusion was not noticed, establishing a steady elevated Ca^2+^ plateau (Figure 5B; overlay of signals).

Taken together, our data suggest that sustained high-frequency stim (1) leads to a rapid GC spike response outage that is accompanied by elevated intercellular Ca^2+^ levels and (2) does not further promote intracellular Ca^2+^ intrusion but facilitates the inhibition of the extrusion clamping of intercellular Ca^2+^ at elevated levels (Figure 5B), and (3) at all applied frequencies post stimulus, the Ca^2+^ level recovers immediately.

## 4. Discussion

Electrical stimulation promotes visual sensation in blind patients; however, at high stim frequencies, artificially induced visual sensations fade rapidly [15,20,21,23]. Experiments on animal retinal explants revealed that the observed vision fading does not occur in the cortex but rather is related to the retinal cells interfaced by the microelectronics, specifically GCs. Generally, the observed response outage of GCs and other neuron types is explained by the desensitization of the membrane channels [7,22,23,24,25]. However, as of yet, unequivocal evidence describing the underlying mechanism for retinal response outage has not been presented.

In our previous study, in stim retinal cells [29], we observed an influx of Ca^2+^ that is dependent on stim pulse strength. Inspired by these findings, we systematically investigated the role of the crucial neuromodulator Ca^2+^ in the electrophysiological response outage. Therefore, we modulated the voltage responsible for the response strength and frequency, allowing us to study the GC response dynamics. In contrast with studies investigating single-pulse applications [29], we applied sustained stim at different frequencies for 90 s, matching the realistic scenario of continuous vision perception.

### 4.1. Switch from Network-Mediated to the Direct Stimulation of Ganglion Cells

Subretinal stim propagating through the retinal network of blind mice is of great advantage, as it mediates advanced retinal responses, such as the differential activation of diverse retinal channels, such as the ON, OFF, and ON–OFF GC subtypes [29]. Hence, in the present study, we evaluated the impact of voltage or frequency modulation on network-mediated responses in the presence of synaptic blockers (LAP-4, CNQX, and verapamil) and a VGNaC blocker (TTX). Our data show that, at stim frequencies <5 Hz, GCs were activated only synaptically, while at ≥5 Hz, GCs were also activated directly over the VGC. Overall, for developing a stim paradigm for subretinal applications, it is key to consider that high voltage at low frequencies or low voltage at high frequencies can penetrate the retinal tissue deeper and in addition activate GCs directly.

### 4.2. High-Frequency Stimulation Electrogenically Modulates the Desensitization of the Ganglion Cells

An unexpected observation was made in Ca^2+^ imaging recordings: With increasing frequencies, the Ca^2+^ signals melted to an elevated Ca^2+^ plateau, while spike responses were absent. These findings correlate with patient reports of experiencing vision fading above 3 Hz stim. Hence, a few questions were raised to assist in understanding the GC response outage dependent on the stim frequency and the role of Ca^2+^.

*(i) What is the correlation between GC spikes and Ca^2+^ signals in our data?* During electrophysiological activity (depolarization), Ca^2+^ enters GCs via the VGNaC (responsible for spike generation (=action potential)) and the additional source of Ca^2+^ intrusion to the VGCC [4]. With increasing frequencies, larger Ca^2+^ amplitudes were measured, indicating the opening of more and more channels facilitating the large Ca^2+^ intrusions. At a stim frequency of 20 Hz, most channels seemed to be open, achieving a maximum response; however, the 50 Hz stim did not yield a significantly more extensive response. After the stim-induced depolarization, the observed Ca^2+^ signals required a relatively prolonged recovery period of 300 ms to become responsive again. Eventually, shortening the resting time (pulse interval) by increased frequencies (10 Hz: 100 ms; 20 Hz: 50 ms; 50 Hz: 20 ms) led to plateau-like elevated intracellular Ca^2+^. In contrast, the refractory period of intrinsically closing VGNaCs roughly lies by half the action potential time (~ 1 ms), in which the balance of ions is maintained, and the neurons become responsive again. To this end, it remains unclear whether the VGNaCs are affected directly or indirectly (e.g., refractory period) by the elevated Ca^2+^ leading to GC response outage, which leads us to our further questions.

*(ii) How is the Ca^2+^ plateau established?* High-frequency stim produced larger Ca^2+^ transient signals within the first few pulses, leading to a steady plateau. With ongoing stim, if Ca^2+^ had entered via VGNaC (synaptically or direct activation), spikes should have been generated, but in the MEA recordings, no spikes were observed. Alternatively, if Ca^2+^ had entered via VGCC, we should have seen Ca^2+^ transients, but this was not the case. Since the Ca^2+^ signal did not increase during the ongoing stim, it can be ruled out that additional Ca^2+^ had entered the cell. It is likely that most of the channels had been opened by the first pulses, establishing the level of the Ca^2+^ plateau.

*(iii) Why is the Ca^2+^ level not decreasing?* Ca^2+^, intruded by electrophysiological actions into cells (spiking or maintaining resting membrane potential), is regulated by several Ca^2+^ extrusion mechanisms [33,34]. Hence, hypothetically, it is conceivable that the observed elevated Ca^2+^ plateau results from an equilibrium of rapid intrusion and extrusion, appearing to be steady. However, we rejected this hypothesis because no spikes (no VGNaC-mediated Ca^2+^ influx) were observed. A successive Ca^2+^ influx mediated by VGCC was also excluded according to the null Ca^2+^ influx rate. Hence, we concluded that the Ca^2+^ extrusion must have been affected electrogenically by the stim, which clamped the initially intruded Ca^2+^ in the cell for the remaining stim period. Higher stim frequencies affected the retinal tissue in depth, stimulating GCs directly (verified by synaptic blockers). First, the electric field excited the GCs but then inhibited the extrusion mechanism electrogenically as a side effect.

Potential Ca^2+^ extrusion candidates that could be electrogenically affected by the electric field [35,36] are charge-dependent mechanisms, such as membrane exchangers (sodium/Ca^2+^ exchanger (NCX)) or channels (voltage-gated and Ca^2+^-modulated potassium (K^+^) channels) [37,38,39]. Otherwise, Ca^2+^ buffering caused by cytosolic Ca^2+^-binding protein [40] stores (ER or mitochondrial stores, [41]) or ATP-driven membrane Ca^2+^ pumps (PMCA, [42]) is unlikely to be affected electrogenically [35,36]. Hence, in our data, the observed initial drop in the Ca^2+^ transient is probably governed by these electrogenically unaffected Ca^2+^ removal mechanisms, eventually reaching their maximum uptake capacity. This, in turn, could explain why a further drop in Ca^2+^ level was not observed. Nevertheless, specific patch clamp recordings could provide further details.

*(iv) How does a response outage occur?* The aforementioned discussion of the evaluated data established that the elevated Ca^2+^ is not extruded during continuous ongoing high-frequency stim. The extrusion is obviously extremely slowed down or nearly blocked. Given that high stimulation frequency can lead to greater cell depolarization [43], the up to 6.5-fold increased calcium levels in our data very likely reflect a strong cell depolarization and shift in the membrane potential toward the Ca^2+^-dominated positive potential. Consequently, it is conceivable that the elevated Ca^2+^ levels impact the regeneration of the resting membrane potential and thereby the generation of further action potentials, resulting in a response outage (no spikes in MEA recordings). The immediate drop in the Ca^2+^ levels after stim (at high and low frequencies) allows for the recovery and regeneration of action potentials. Hence, we concluded that high-frequency stim (≥5 Hz) electrogenically prevented cellular Ca^2+^ extrusion and clamped the intracellular Ca^2+^ levels at remarkably high levels, with strong electrophysiological implications for the further generation of GC spikes. This electrogenic blockage might also affect dendrites and synapses throughout the retinal neuronal network [37]. A natural GABAergic or glycinergic response inhibition is inconceivable since synaptic inhibition would not lead to elevated Ca^2+^ levels—the inhibitory pathway is probably equally stim. In addition, Ca^2+^, Na^+^, and K^+^ play a significant role in the maintenance of electrophysiological resting membrane potential [4,44,45]. Their contribution could be investigated using methods of patch clamp recordings to narrow down the contribution of ions, channels, or exchangers.

*(v) What is the difference in outcome between high-rate stimulations from our study and those of others?* Comparing previous findings, Weiland, Walston, and Humayun (2016) [24] have concluded that high-frequency stimulation leads to substantial GC response decay (>25 Hz) and even response outage (>50 Hz), within the very first 10 stimulation pulses; in our approach, up to 45–4500 pulses were applied using 0.5–50 Hz stimulation during a sustained stimulation period of 90 s. Considering the 16 Hz stimulation conducted by Freeman and Fried (2011) [23], they investigated the responses to 10 sequential stimulation pulses (total stimulation period of 625 ms), which is comparable in our recordings to the “initial phase” stimulation of the total 90 s recording period (Figure 3). Within this short stimulation period, they reported a drop in the GC spike in the first stimulation from ~10 to 0–2 spikes in the tenth stimulation, which is in line with our MEA and Ca^2+^ data (Figure 5, <500 ms): The strongest Ca^2+^ transient (intrusion) was observed for the very first stimulation pulses despite the frequency (20 Hz: 10 pulses in 500 ms, and 50 Hz: 25 pulses in 500 ms). Within an earlier study, Sekirnjak et al. (2006) [46] reported robust GC responses to 100 and even 300 Hz stimulation. Thereby, they applied paired pulses with a pause interval of 500 ms. It is worth noting that, in their work, the notation high-frequency refers to the time interval of the paired pulses and not to the pause interval. Considering the pause interval of 500 ms, which is most crucial (the intervals of the two stimulation pulses were only 10 and 3 ms), these data correspond to our lower stimulation frequencies (~0.5 Hz) and are in line with our outcome, confirming that a ~ 300 ms interval is required for Ca^2+^ recovery and the regeneration of spike activity.

Overall, previous findings do not contradict our findings; however, our approach goes beyond the scope of previous investigations in terms of stimulation duration (90 s), stimulation frequency, sustained application, and the discrimination of different stimulation phases (initial, middle, and recovery). The use of Ca^2+^ imaging recordings led us to the most interesting observation: as long as the Ca^2+^ levels were elevated within the GCs, no further Ca^2+^ transients were observed in Ca^2+^ data, and a GC spike response outage was observed in MEA data.

### 4.3. Implications of Electrical-Stimulation-Mediated Modulation of Cellular Ca^2+^ Dynamics for the Development of Strategies for Electrical Implants

The presented Ca^2+^ imaging recordings of GCs in conjunction with different pharmaceuticals unequivocally revealed that the applied stim (voltage and frequency) did not electroporate the retinal cells; the GC activity was based on neurotransmitter transmission or voltage-sensitive channels.

The cellular recording resolution of Ca^2+^ imaging allowed us to conclude that not all GCs responded to the same stim and not all applied voltages produced GC responses. These findings might guide efforts investigating the differential activation of GCs, for example, as established by Baden et al. (2016) [32] for light stim. Furthermore, future studies considering mathematical or computational models inferring spike responses from Ca^2+^ imaging [47,48,49] for the development of stim paradigms [27,50,51,52], shall not only consider the primary excitatory effect of electrical stimulation on neurons, but also with care the secondary effects like response outage and ion extrusion as described in the presented study.

Seeking stimulation paradigms to drive the different retinal channels independently is the ultimate goal of artificial vision research. Therefore, different parameters are applied in variations to obtain new insights. In schemes of subretinal stimulations (outer retina), Jensen and Rizzo (2009) [53] suggested that a biphasic anodic-first stimulation might be a better choice than monophasic cathodic and monophasic anodic stimulation. In contrast, cathodic-first biphasic stimulation was used successfully in subretinal retinal implants [9] promoting visual sensations in blind patients. Thereby, the voltage of both phases was equal, thus achieving a balanced charge. Later, Haq et al. (2018) [29] successfully demonstrated the network-mediated activation of different GC types using monophasic anodic stimulation in subretinal configuration, interfacing the residual light-insensitive degenerated photoreceptors. Yet, complementing and extending the range of research in this field, we tested for the first time biphasic cathodic-first stimulation in a subretinal configuration in addition to biphasic anodic-first stimulation, both in an unbalanced manner (keeping the anodic phase static and modulating only cathodic phase). In addition, the distance of the reference electrode was considered (nearby and far). Our data showed that a biphasic cathodic-first stimulation was most effective in activating the GCs via the retinal network (with a nearby reference electrode) and that not all voltage combinations activated the cells with the same strength. Our findings might seemingly conflict with other findings (different response strengths), but most of the studies were performed using electrical MEA recordings; in contrast, our work was performed using Ca^2+^ imaging. Thus, our study enriches research in this field by highlighting the role of ions, particularly Ca^2+^, in shaping physiological responses.

Different strategies have been suggested for overcoming the phenomenon of stim-induced vision fading by avoiding cell desensitization. Sekhar et al. (2016) [54], Li et al. (2022) [55], and Chenais et al. (2021) [56] modulated the applied voltage randomly and activated the electrodes line-wise or randomly. In principle, these findings indirectly support our results. It appears that the randomly applied low voltages allowed intracellular Ca^2+^ to recover (comparable to our data, not all subvoltages excite cells; Figure 2), and the subsequent high-voltage stim clamped cellular Ca^2+^ again (comparable to high-frequency stim in our data; Figure 3). However, the random stim application [54,56] would not translate the light intensity perceived by implants’ photodiodes into an intensity–stimulus relation, for example, considering contrast and day/night light settings. In contrast, within an applicable solution for retinal implants, enhanced high temporal and spatial resolution responses have been achieved recently [25]. Thereby, six contiguously arranged electrodes (hexagons) were activated alternatingly, resembling biomimetically saccadic eye movements. The predictive “jump” of the activated electrode gave cells in contact with inactive electrodes enough time to recover from the previous stim (estimated recovery time ~ 300 ms). Our data indicate that the sequential electrode jumps [25] facilitated the required recovery time for cells to extrude Ca^2+^ to a level that allowed for the regeneration of spikes.

In addition to the electrophysiological impact, the response outage and sustained elevated Ca^2+^ baseline due to successive high-frequency stim could also cause secondary neuronal damage by activating Ca^2+^-triggered cell degenerative pathways [57].

## 5. Conclusions

Our study highlights the paramount role of the neuromodulator Ca^2+^ in GC response outages that are dependent on stim frequency. The newly established protocol, stimulating blind mouse retinas in a subretinal configuration and recording GC responses via Ca^2+^ imaging and MEA, allowed us to elucidate the Ca^2+^ dynamics and determine the underlying mechanism.

The combination of stim parameters (biphasic pulse, cathodic-first stimulation with a near reference, and stim frequency) influenced the response strength and stim depth, thereby regulating cellular Ca^2+^ intrusion. On the other end, the disruption of Ca^2+^ extrusion was found to be responsible for the GC response outage.

The temporal correlation of GC Ca^2+^ responses and spike trains revealed that, at high-frequency stim (>3 Hz sustained application), a GC spike response outage occurred, while at the same time, the intracellular Ca^2+^ level was remarkably elevated. We concluded that high-frequency stim prevented electrogenically cellular Ca^2+^ extrusion and thereby clamped the intracellular Ca^2+^ at high levels, in turn contributing to the electrophysiological GC spike outage for the remaining duration of the sustained stim.

These findings are of profound interest for studies optimizing electrical stimulation paradigms for artificial vision to prevent vision fading and, overall, for applications of electrical neural interfacing.

## Data Availability

The data presented in this study are available in the article.

## Supplementary Materials

The following supporting information can be downloaded at: https://www.mdpi.com/article/10.3390/bioengineering10101208/s1, Table S1: Statistical analysis of Figure 4.

## Abbreviations

The following abbreviations are used in this manuscript:

ACSF	Artificial cerebrospinal fluid
BC	Bipolar cells
Ca^2+^	Calcium
CB OFF	OFF-type cone bipolar cell
CB ON	ON-type cone bipolar cell
CICR	Calcium-induced calcium release
CNQX	6-Cyano-7-nitroquinoxaline-2,3-dione
ctr	Control
elec	Electrode
ER	Endoplasmic reticulum
GC	Ganglion cell
Hz	Hertz
iGluR	Ionotropic glutamate receptors
K^+^	Potassium
kHz	Kilo hertz
L-AP4	L-2-amino-4-phosphonobutyric acid
MEA	Multielectrode array
mGluR6	Metabotropic glutamate receptor 6
mM	Milli molar
Na^+^	Sodium
NCX	Sodium/calcium exchanger
OGB-1	Oregon Green 488 BAPTA-1
PMCA	Plasma membrane calcium ATP-pump
*rd1*	C3H/rd1 (C3H Pde6b^rd1/rd1^)
ref	Reference
SEM	Standard error mean
stim	Electrical stimulation
TTX	Tetrodotoxin
V	Volt
VGC	Voltage-gated channels
VGCC	Voltage-gated calcium channels
VGNaC	Voltage-gated sodium channels
δ	Delta (difference)
τ	Tau
µV	Microvolt
∆	Rate (change over time)
