# Supplementary material for: Artificial Vision: The High-Frequency Electrical Stimulation of the Blind Mouse Retina Decay Spike Generation and Electrogenically Clamped Intracellular Ca2+ at Elevated Levels
Source: Bioengineering (Basel). 2023 Oct 16;10(10):1208. doi: 10.3390/bioengineering10101208 (PMC10604554; doi:10.3390/bioengineering10101208)
Supplement: Supplementary file 1 [file bioengineering-10-01208-s001.zip › bioengineering-2587155 - supplementary.pdf]

Table S1. Statistical analysis of Figure 4. Statistical analysis was performed to compare control frequency-ramp in (A: amplitude and B: level in middle phase) using the Wilcoxon-Mann-Whitney test. To access the impact of drugs in C and D, one-way ANOVA followed by the Dunnett´s multiple comparison test was used. Significance levels were: not significant (n.s.), $^{*}P < 0.05$, $^{**}P \leq 0.01$ and $^{***}P \leq 0.001$.

**A) Statistical analysis of control amplitude in dependence to frequencies (Fig. 4A, ctr)**						
**Frequency [Hz]**	0.5 vs 1.5	1.5 vs 3.0	3.0 vs 5.0	5.0 vs 10	10 vs 20	20 vs 50
**p-Values**	0.99 (n.s.)	1.0 (n.s.)	0.82 (n.s.)	0.99 (n.s.)	1.04E-06 (***)	0.98 (n.s.)

**B) Statistical analysis of control calcium levels in dependence to frequencies (Fig. 4B, ctr)**						
**Frequency [Hz]**	0.5 vs 1.5	1.5 vs 3.0	3.0 vs 5.0	5.0 vs 10	10 vs 20	20 vs 50
**p-Values**	0.99 (n.s.)	0.04 (*)	0.02 (*)	0.002 (**)	0.004 (**)	0.97 (n.s.)

**C) Statistical analysis of amplitude in dependence to frequencies and drugs (Fig. 4A)**							
	0.5 Hz	1.5 Hz	3.0 Hz	5.0 Hz	10 Hz	20 Hz	50 Hz
ctr - (LAP4 + CNQX)	1.68E-06 (***)	1.47E-06 (***)	1.47E-06 (***)	2.46E-08 (***)	2.11E-07 (***)	1.21E-05 (***)	1.64E-07 (***)
ctr - TTX	6.43E-07 (***)	3.33E-06 (***)	3.33E-06 (***)	5.34E-08 (***)	1.45E-08 (***)	3.40E-07 (***)	2.56E-08 (***)
ctr - Verapamil	4.27E-06 (***)	6.75E-06 (***)	6.75E-06 (***)	1.24E-07 (***)	4.42E-08 (***)	1.11E-06 (***)	1.22E-07 (***)
(LAP4 + CNQX) - TTX	0.99 (n.s.)	0.84 (n.s.)	0.84 (n.s.)	0.43 (n.s.)	0.04 (*)	0.04 (*)	0.43 (n.s.)
(LAP4 + CNQX) - Verapamil	1.00 (n.s.)	1.00 (n.s.)	1.00 (n.s.)	1.00 (n.s.)	0.04 (*)	0.03 (*)	0.04 (*)
TTX - Verapamil	1.00 (n.s.)	0.97 (n.s.)	0.97 (n.s.)	0.92 (n.s.)	0.97 (n.s.)	1.00 (n.s.)	0.03 (*)

**D) Statistical analysis of calcium levels in dependence to frequencies and drugs (Fig. 4B)**							
	0.5 Hz	1.5 Hz	3.0 Hz	5.0 Hz	10 Hz	20 Hz	50 Hz
ctr - (LAP4 + CNQX)	0.39 (n.s.)	0.06 (n.s.)	1.83E-06 (***)	4.75E-05 (***)	3.25E-07 (***)	2.70E-07 (***)	2.80E-06 (***)
ctr - TTX	0.01 (**)	0.71 (n.s.)	2.80E-06 (***)	8.34E-07 (***)	4.39E-08 (***)	2.09E-08 (***)	4.39E-08 (***)
ctr - Verapamil	0.24 (n.s.)	0.51 (n.s.)	1.15E-05 (***)	3.02E-06 (***)	2.01E-07 (***)	9.42E-08 (***)	3.02E-06 (***)
(LAP4 + CNQX) - TTX	0.10 (n.s.)	0.36 (n.s.)	0.97 (n.s.)	0.03 (*)	0.04 (*)	0.01 (**)	0.03 (*)
(LAP4 + CNQX) - Verapamil	0.99 (n.s.)	0.89 (n.s.)	1.00 (n.s.)	0.04 (*)	0.04 (*)	0.02 (*)	0.03 (*)
TTX - Verapamil	0.48 (n.s.)	0.99 (n.s.)	1.00 (n.s.)	1.00 (n.s.)	1.00 (n.s.)	1.00 (n.s.)	1.00 (n.s.)
